# Cultivar ideotype for intensive olive orchards: plant vigor, biomass partitioning, tree architecture and fruiting characteristics

**DOI:** 10.3389/fpls.2024.1345182

**Published:** 2024-01-25

**Authors:** Adolfo Rosati, Andrea Paoletti, Enrico Maria Lodolini, Franco Famiani

**Affiliations:** ^1^ Consiglio per la Ricerca in Agricoltura e l’analisi dell’Economia Agraria (CREA), Centro di Ricerca Olivicoltura, Frutticoltura e Agrumicoltura, Spoleto, Italy; ^2^ Dipartimento di Scienze Agrarie, Alimentari e Ambientali, Università Politecnica delle Marche, Ancona, Italy; ^3^ Dipartimento di Scienze Agrarie, Alimentari e Ambientali, Università degli Studi di Perugia, Perugia, Italy

**Keywords:** breeding, cultivar, fruit trees, harvest index, sink, source, training, yield

## Abstract

In order to achieve higher and earlier yield, modern olive orchards are increasingly intensified, with tree densities up to > 1500 trees hectare^-1^. With increasing tree densities, individual-tree canopy volume must be proportionally reduced. Not all cultivars are adaptable to high and very high orchard densities, because of excessive vigor and/or insufficient bearing when the canopy is pruned to a small volume. However, what makes an olive cultivar suitable for intensive and super intensive orchards is not clear. Recently, few studies have addressed this topic, suggesting that tree architecture and early bearing are essential traits. Yet, what architectural and productive features are important, how they work and whether they are interrelated remains elusive. This review summarizes and interprets the literature on olive, as well as the more abundant literature available for other fruit species, aiming to provide a comprehensive knowledge framework for understanding how tree architectural characteristics, plant vigor, and fruiting vary across olive genotypes, and how they are interconnected. It is concluded that, among the architectural characteristics, greater branching and smaller diameters of woody structures are particularly important features for cultivar suitability to intensive and super intensive olive orchards. Greater branching allows to produce more fruiting sites in the small volume of canopy allowed in these systems. It also reduces investments in woody structures, liberating resources for fruiting. Additional resources are liberated with smaller structure diameters. Greater branching also increases resources by increasing biomass partitioning into leaves (i.e. the photosynthetic organs), relative to wood. Since yield is affected by the competition for resources with vegetative growth, reducing resource investments in woody structures and/or increasing resource directly, increases yield. Yield, in turn, depresses vegetative growth, reducing vigor and the need for pruning. High yields also produce short shoots which have relatively greater investments in leaf mass and area, and lower in the woody stem, making them more suitable than long shoots to support concurrent fruit growth. This single framework of interpretation of how the different architectural and fruiting characteristics work and interact with one-another, will provide guidance for cultivar selection and breeding for intensive and super intensive olive orchards.

## 1 Introduction

Olive growing has been shifting from traditional low-density orchards to intensive orchards, including super intensive (or super high density, SHD) orchards (up to > 1500 trees hectare^-1^), which are still a minority but are increasing (Guerrero-Casado et al., 2021). As the olive system intensifies, low vigor and early and abundant bearing are essential traits (Tous et al., 1999; Rallo et al., 2007), indispensable to make SHD orchards economically viable (De Benedetto et al., 2003). However, despite much research on tree vigor and dwarf cultivars (Barranco, 1997; Sonnoli, 2001; León Moreno, 2007), few cultivars appear to combine such traits. In particular, only few cultivars are suitable for SHD orchards, where canopy volume is necessarily limited by the close spacing and the size of the harvesting machine (i.e. an over-the-row machine). Most other cultivars “escape” quickly from this small volume, and therefore require heavy pruning (Vivaldi et al., 2015). This stimulates strong vegetative regrowth, reducing fruiting (Jerie et al., 1989). In fact, cultivar response to different pruning types is an essential characteristic for olive cultivar suitability to SHD orchards (Vivaldi et al., 2015). The cultivars most used for SHD orchards are Arbequina, Arbosana and Koroneiki (Tous et al., 2014) and few more recent ones are being considered, like Chikitita (Rallo et al., 2008), Oliana (Butler, 2014) and Lecciana (Camposeo et al., 2021). These cultivars are thought to have lower vigor and to be earlier bearing and less alternate bearing than other cultivars (Moutier et al., 2004; Moutier, 2006; Tous et al., 2006; Camposeo et al., 2008; Moutier et al., 2008; Godini et al., 2011; Caruso et al., 2014; Farinelli and Tombesi, 2015; Díez et al., 2016). However, only few studies report detailed data on plant vigor, biomass partitioning, tree architecture and fruiting characteristics that distinguish such cultivars from those that are not suitable to SHD orchards. In this review, the literature available on all these aspects is summarized and interpreted.

## 2 Plant vigor, biomass partitioning and competition between vegetative and reproductive growth

Plant vigor depends on the cultivar (genotype) and its interaction with the environment, including agronomic management. When new orchards are established, plant density is decided based on plant vigor (Del Río et al., 2002). Dwarfing is often induced by grafting on dwarfing rootstocks (Donadio et al., 2019). Dwarf trees allow increased orchard density due to their low vigor and limited crown size (Tombesi and Farinelli, 2016). Smaller trees and higher density allow early bearing and early entrance of the orchard into full production (Koumanov and Tsareva, 2017).

In cultivated plants, the ratio of biomass invested in the harvested part over the total biomass of the plant is called harvest index (HI), and expresses the efficiency of dry matter partitioning (Donald, 1962). In tree crops, the HI is often indicated as harvest increment (HIn), which is defined as the ratio between the harvested part and the total above-ground biomass increment, over one year or a longer period (Cannell, 1985). Current fruit tree cultivars have higher HI than their wild counterparts (Patrick, 1988). HI may be 0-20% in forest trees, and up to 75-80% in cultivated species (Cannell, 1985; Fischer et al., 2012). The higher productivity of cultivated plants results from their greater HI, while their photosynthetic rates do not differ (Loomis, 1983). This implies that tree growth is driven by the competition for assimilates between vegetative organs and reproductive structures (Kramer and Kozlowski, 1979; Spurr and Barnes, 1980; Grossman and DeJong, 1995a; Grossman and DeJong, 1995b).

Reducing tree growth, by water stress (Mitchell et al., 1989), dwarfing rootstocks (Preston, 1958; Avery, 1970), containing roots with drip irrigation (Mitchell and Chalmers, 1983), pruning (Geisler and Ferree, 1984), shoot removal and/or chemical control of vegetative growth (Williams et al., 1986; Rugini and Pannelli, 1993; Mulas et al., 2011), can all increase fruit yield, suggesting competition between vegetative growth and fruit production.

In fact, fruit growth requires abundant photosynthates and trunk, branch, and, especially, root growth decreases at increasing fruit load (Lakso and Flore, 2003). In fruit trees, fruit and vegetative growths overlap for large periods, inducing strong competition for resources (Forshey and Elfving, 1989; DeJong, 1999; Wünsche and Ferguson, 2005). This competition is well documented for adult trees of many species (Stevenson and Shackel, 1998; Costes et al., 2000; Berman and DeJong, 2003), including for olive (Monselise and Goldschmidt, 1982; Rallo and Suárez, 1989; Obeso, 2002; Connor and Fereres, 2005; Lavee, 2007; Dag et al., 2010; Castillo-Llanque and Rapoport, 2011). The effects of this competition are even more dramatic in young fruit trees, where preventing fruit set greatly increases tree growth (Chandler and Heinicke, 1926; Verheij, 1972; Forshey and Elfving, 1989; Embree et al., 2007). This is the case also for young olive trees (Rosati et al., 2018a; Rosati et al., 2018b; Paoletti et al., 2021). In fact, deflowering has been proposed as an effective tool to accelerate the growth of young olive trees, thus shortening the unproductive phase in new orchards (Famiani et al., 2022).

Achieving a balance between vegetative growth and fruiting is a research priority in horticulture, given that further gains in productivity are thought to be obtainable by reducing the vegetative growth necessary to allow fruit growth (Elfving, 1988). Yet, a sufficient development in vegetative organs is necessary to intercept radiation and to absorb water and nutrients. In fruit trees, this competition has been studied mostly on plant parts, because it is easier than working at the whole-tree level. Most frequently, individual shoots have been studied (e.g. Rallo and Suárez, 1989; Acebedo et al., 2000), comparing shoot growth of fruiting and non-fruiting trees (Cimato and Fiorino, 1986; Proietti and Tombesi, 1996). Less frequently authors have considered populations of modules (Hasegawa and Takeda, 2001), or whole branches (Castillo-Llanque and Rapoport, 2011). Only a whole-tree approach, however, can provide quantitative descriptions of the competition between vegetative growth and fruit growth. Such approaches are infrequent, mostly found in older works (Verheij, 1972; Forshey and Elfving, 1989), reporting defruiting or rootstocks effects. Furthermore, even in these works, vegetative growth was compared between “off” and “on” years/trees/shoots, not allowing to provide quantitative relationships between vegetative growth and fruiting at the whole-tree level, as was done more recently in young olive trees (see below).

There is scant data on the HI for adult olive trees. In adult Arbequina trees, Villalobos et al. (2006) reported an HI of 50%, considering only the aerial parts, which corresponds to an HI of 35% if the aerial parts are assumed to be 70% of the total biomass increment.

Quantitative data on biomass partitioning in young olive trees is also scarce (Scariano et al., 2008; Di Vaio et al., 2012; Di Vaio et al., 2013). Recently, however, it has been reported that deflowering (i.e. preventing fruit set) young olive trees, strongly increased vegetative growth, and eliminated cultivar differences in vigor between Frantoio and Arbequina (high and low vigor, respectively), suggesting that differences in cultivar vigor arise from differences in biomass partitioning into fruit (Rosati et al., 2018a; Rosati et al., 2018b; Paoletti et al., 2021). This work was done at the whole-tree level and thus provided quantitative relationships between fruit production and reduction in vegetative biomass accumulation. Tree growth was inversely related to fruiting efficiency also across 12 cultivars (Rosati et al., 2017). All of this demonstrates that resource competition is a strong determinant of tree growth in young olive trees, and suggests a source limitation to tree growth (Rosati et al., 2018b).

### 2.1 Biomass partitioning into flowers

Like fruit, flowers subtract resources from the vegetative growth. Paoletti et al. (2021) calculated the HI for flowers by considering the inflorescences’ biomass in relation to the total biomass increase. The flower HI was already high in 3-4-year-old Arbequina, reaching 8-9% in fruiting plants, and 16-18% in deflowered plants. Higher values in deflowered plants were related to the fact that preventing fruit formation increased resource availability. This stimulated both vegetative growth and greater flower induction and differentiation the following years resulting in more flower production than in fruiting trees. Famiani et al. (2019) reported that, in adult Frantoio trees, the cost of flowering (i.e. the proportion of biomass invested in inflorescences) was about 17% of the total biomass of inflorescences + fruit. Assuming an HI of 50% in adult olive trees (Villalobos et al., 2006), this value (17%) corresponds to 8.5% of the biomass increment of the whole tree, a value nearly identical to that found by Paoletti et al. (2021) in young fruiting trees. Considering that inflorescences develop over a much shorter period, compared to the fruit, the rate of biomass allocation to the inflorescences is similar to the later rate of allocation to the fruit (Famiani et al., 2019), implying a considerable effort in inflorescence formation.

### 2.2 Partitioning among vegetative sinks

In olive, Paoletti et al. (2021) found substantial cultivars differences in the distribution of vegetative biomass in young trees, especially at the leaf level: while in Arbequina leaf dry matter represented about 40% of total biomass during the first four years after transplanting, in Frantoio it decreased from 40 to 20%. Similar results were reported for 3-year-old Nocellara del Belice, with about 30% biomass in leaves (Scariano et al., 2008), but data did not include the trend over the years. This value is similar to that reported by Paoletti et al. (2021) for Frantoio at the same 3-year-old age. Di Vaio et al. (2012) found that both leaves and roots represented 28% of the total biomass in 1-year-old Leccino trees. This value is respectively lower (for leaves) and higher (for roots) than found by Paoletti et al. (2021) for trees of the same age. These differences could be related to varietal effects or different trial conditions. Di Vaio et al. (2013) reported data on the biomass composition of 2-year-old trees of Leccino (high vigor) and Racioppella (low vigor), subjected to different irrigation regimes. Although the percentage composition of the plant components was not reported, it could be calculated from the absolute values. In well-irrigated Leccino trees, leaves amounted to 25% of total dry biomass in the first year, but the percentage decreased to 16% in the following year. In less irrigated plants, the values were, respective, 22% and 14%. Results were similar for Racioppella. The fraction of root biomass increased over the two years, from 16% to 28% in Leccino (well-irrigated) but less so, from 30 to 34%, in Racioppella. Irrigation levels are known to affect dry matter partitioning in olive (Xiloyannis et al., 1999; Dichio et al., 2002; Bacelar et al., 2007; Di Vaio et al., 2012; Di Vaio et al., 2013). Cultivar effects have been reported as well (Tognetti et al., 2002; Di Vaio et al., 2013). Overall, the published data appear to indicate a general trend in decreasing partitioning towards leaves and increasing partitioning towards roots in most cultivars, with the exception of Arbequina where partitioning towards leaves does not decrease, at least in the first four years of age (Paoletti et al., 2021). These authors also found that partitioning towards branches increases over the years for both cultivars tested, whether at the expense of leaves (as in Frantoio) or of other plant parts (as in Arbequina). Thus, relative to other cultivars, Arbequina appears to maintain greater biomass investments in leaves, therefore greater leaf area, relative to tree size and age (Rosati et al., 2018c). This is advantageous in young trees, because it allows higher light interception and thus faster growth (Rosati et al., 2018c).

Paoletti et al. (2021) reported that the canopy to root ratio increased with age in Arbequina compared to Frantoio, confirming previous reports that this ratio differs among cultivars (Di Vaio et al., 2012; Di Vaio et al., 2013). Furthermore, in Arbequina this ratio had higher values in fruiting trees, compared to defruited ones Paoletti et al. (2021), confirming that roots are weaker sinks compared to other plant organs, particularly fruit. Therefore, partitioning towards roots is increasingly reduced at increasing fruit load (Lakso and Flore, 2003). Paoletti et al. (2021) also found that the bearing branches (i.e. stem and leaves of shoots from previous and current year) to structural wood (branches, trunk and root) biomass ratio was about 1 gg^-1^ in fruiting Arbequina trees. That means that for one gram of dry matter partitioned to non-productive structures, there was one gram partitioned to productive structures. In Arbequina the average ratio for the different fruit load treatments, was almost double the Frantoio’s values. Higher values of the bearing branches to structural wood biomass ratio are considered functional to increase productivity, as they allow the plant to save resources that would be spent in unproductive structures (Rosati et al., 2013; Rosati et al., 2018c). Trunk and branches, in fact, are not directly productive structures, as they do not photosynthesize, although they are still indispensable, allowing leaf distribution in space, thus maximizing light interception. They also bring water and nutrients from the soil to the leaves. Similar considerations apply to the root system: while not directly productive, it is indispensable to absorb and transport water and nutrients, and to anchor the plant to the soil.

## 3 Is tree growth source or sink limited?

Evidence for a source limitation to plant growth appears to contrast with previous findings that growth is, instead, sink limited (Fatichi et al., 2014; Palacio et al., 2014) and that photosynthesis is controlled by sink availability (Boussingault, 1868; Gucci et al., 1991; Gucci et al., 1995; Iglesias et al., 2002). In fact, at times photosynthesis is higher when fruit is present, suggesting sink limitation in the absence of fruit (sinks). This usually occurs when girdling or other extreme treatments are applied in the absence of fruit (Quentin et al., 2014), or with external applications of sugar (Iglesias et al., 2002; Ribeiro et al., 2017), at elevated CO_2_ concentrations (Ainsworth et al., 2004), in rooted leaves without other growing organs (Sawada et al., 1986), or with continuous light (Sawada et al., 1986; Layne and Flore, 1995).

This photosynthetic down regulation is probably linked to the accumulation of photosynthesis end-products, such as starch (Paul and Foyer, 2001) or soluble sugars (Franck et al., 2006; McCormick et al., 2006), which cannot be quickly exported due to the lack of sinks (Ainsworth et al., 2004). In some cases, the photosynthetic down-regulation may be observed only when some nutrient deficiency occurs (Pieters et al., 2001; Pan et al., 2017) or only in the short term (Gucci et al., 1991; Pan et al., 2017), while in well-nourished plants, or in the long term, the plants resume sink activity and the down regulations vanishes. All of this might suggest that sugar accumulation in the leaves, due to insufficient export to limited external sinks might cause the negative feedback to photosynthesis. However, it has been recently found that this downregulation begins before sugar accumulation become substantial, suggesting that, rather than the sugar content, the signal for photosynthesis downregulation might be the change in sugar turnover (Nebauer et al., 2011). Paul and Foyer (2001) reviewed the subject and concluded that “photosynthesis responds to and is controlled by whole plant source-sink balance, controlled by whole plant nutrient balance, principally by the carbon and nitrogen status”.

In other studies, photosynthesis was not found to be affected by source-sink manipulations (Egli and Bruening, 2003; Matsuda et al., 2011). In other studies yet, photosynthesis was found to decrease, rather than increase, when fruit was present, and this was attributed to the competition for nutrient, in situation of nutrient deficiency (Zhang et al., 2013; Saa and Brown, 2014; Bote and Jan, 2016). Under water, temperature, or other stress, evidence is increasing that the stress acts directly on the sinks, reducing their sink activity (i.e. growth) before photosynthesis is affected, explaining why sugars concentrate in many stress situations (Muller et al., 2011; Fatichi et al., 2014; Palacio et al., 2014).

In addition to sink-regulation, photosynthesis can be downregulated via stomatal closure (DeJong, 1986), which, in turn, increases leaf temperature and reduces transpiration (Li et al., 2007). This occurred in defruited trees, which had many more new and more vigorous shoots, implying higher transpiration, thus justifying stomatal closure and limitation to leaf photosynthetic rates (though not of whole-canopy photosynthesis). Li et al. (2007) found similar results, additionally reporting that the stomatal limitation increased leaf temperature, further down-regulating photosynthesis. Similar conclusions were reached by Rosati et al. (2018b).

Given the evidence for both sink and source limitation to photosynthesis, it is reasonable to assume that plant growth can be both sink and/or source limited, depending on the situation (Paul and Foyer, 2001; Ainsworth et al., 2004). However, under natural conditions and in the absence of stress, photosynthesis downregulation is unlikely (Stitt, 1991).

In olive, under natural conditions leaf photosynthesis was not downregulated in the absence of fruit (Proietti, 2000; Proietti et al., 2006), although downregulation occurred with girdling, but when no fruit was present (Proietti and Tombesi, 1990). Haouari et al. (2011) reported that photosynthesis was reduced when no fruit was present, however shoot tips had been removed, artificially enhancing sink limitation. It may be concluded that, in olive, in the presence of active sinks, photosynthesis downregulation via accumulation of photosynthates is unlikely. Removing fruit may lead to some stomatal limitation to leaf photosynthetic rates when leaf area is increased due to increased source availability for vegetative growth.

## 4 Tree architecture

Leaves are the main contributors of autotrophic activity in most plants and crops. However, to explore the environment and find sufficient light, leaves need supporting structures. In trees, these are woody structures, including shoot stems, branches, trunk, and roots. These structures are necessary, yet unproductive, thus representing a “cost”. In natural environment, with unselected trees, this cost is high because the trees need to invest more in woody structures to compete for light with other trees. In cultivated situations, the competition for light is reduced by orchard management and selected fruit tree cultivars are smaller (i.e. have lower growth rates) and invest proportionally less biomass in supporting woody structures, and proportionally more in leaves and fruit (Cannell, 1985; Patrick, 1988; Obeso, 2002). The higher productivity of modern cultivars is achieved mostly in this way (Forshey and McKee, 1970; Archbold et al., 1987), while photosynthetic abilities have not changed (Evans, 1976; Loomis, 1983). Hence, understanding the biomass partitioning into woody structures, fruit and leaves is fundamental for yield improvements.

Plant architecture is an important topic in horticulture (Hallé and Oldeman, 1970; Oldeman, 1974; Hallé et al., 1978), particularly in tree crops. Plant architecture results from the temporal and spatial arrangements of the plant parts, and results from morphological traits of shoots and branches (Lauri, 2007). The plant develops its shape through a specific growth pattern or “architectural model”, which represents its basic growth strategy. Analyzing a plant’s architecture is important in order to understand its growth, branching pattern and productivity, and to develop crop models. The architectural parameters more often studied are growth, branching, the lateral vs. apical position of reproductive structures, and the morphological differentiation of axes, (Hallé and Oldeman, 1970; Hallé et al., 1978; Barthélémy et al., 1997; Caraglio and Barthélémy, 1997; Barthélémy and Caraglio, 2007).

Both endogenous (i.e. genetic) and exogenous (i.e. environmental) factors affect the plant’s architecture (Hallé et al., 1978). Genetic factors change across cultivars within a species. In apple, for instance, where studies on tree architecture abound (Costes et al., 2006), traits such as branching density (Lespinasse and Delort, 1986; Forshey et al., 1992), branching frequency (Lauri, 2007) and flowering abundance on lateral shoots (Lauri and Lespinasse, 2001), all vary largely across cultivars, although they are also modulated by environmental variables like temperature, for instance (Cook and Jacobs, 1999; Labuschagné et al., 2003; Naor et al., 2003). In apple and other species, the proportion of short and long shoots also varies across genotypes (Lespinasse and Delort, 1986).

Rootstocks can affect the plant size and branching pattern, thus they are used to control tree size, allowing higher tree densities in modern orchards (Costes et al., 2006). Dwarfing rootstocks increase biomass partitioning into fruit and decrease it into wood, thus increasing the HI, as found in pear, apple, and cherry (Atkinson and Else, 2003). However, this strategy does not work in olive where grafting with dwarfing rootstocks has failed to induce earlier and higher yields in vigorous cultivars (Baldoni and Fontanazza, 1990; Troncoso et al., 1990; Pannelli et al., 1992; Barranco, 1997; Pannelli et al., 2002). This is probably related to the fact that, in olive, the formation of sufficiently long 1-year-old shoots is necessary for flowering and fruiting, while other fruit species, like apple (Costes et al., 2006), can produce flowers and fruit on short shoots (brachyblasts) such as bourses and spurs, allowing abundant fruiting with little vegetative growth. The olive does not have brachyblasts (Castillo-Llanque and Rapoport, 2011), and fruit set only occurs on sufficiently long 1-year-old shoots (macroblasts). Therefore, an abundant bloom (and yield) is possible only with sufficient previous-year vegetative growth. Hence, vigor reduction reduces fruiting sites and potential yield (Rosati et al., 2013). Thus, agronomical practices reducing vigor are unlikely to increase yield efficiency. In fact, using root constriction techniques, while reducing tree growth and size in olive, does not improve fruiting efficiency, leaving cultivar differences in fruiting efficiency unaltered (Paoletti et al., 2023). Therefore, in olive, while removing fruit increases vigor, reducing vigor through agronomical practices does not increase fruiting, suggesting that abundant fruiting is the cause and not the consequence of low vigor.

Since dwarfing rootstocks are unlikely to increase yield and yield efficiency in olive, the preferred way to increase orchard density has been the selection suitable cultivars. Arbequina, Arbosana and Koroneiki (Tous et al., 2014), are the most used cultivars worldwide for SHD orchards, and are considered low-vigour, early bearing, and with low alternate bearing (Moutier et al., 2004; Moutier, 2006; Tous et al., 2006; Camposeo et al., 2008; Godini et al., 2011). However, until recently, no studies reported detailed data on plant vigor and on which tree architectural characteristics might contribute to distinguish these cultivars from others, less suitable for SHD orchards. In other fruit species, such as pear and apple, plant growth and reproduction (i.e. yield) are closely related to the morphology of the axes and the axes’ position within the canopy or, in other words, to the architecture of the plant (Normand et al., 2009). In olive, the fruit distribution in fruiting shoots has been studied across different cultivars (Moutier et al., 2004). Other architectural features have been studied in non-fruiting young seedlings (Hammami et al., 2011; Hammami et al., 2021). Detailed studies on the genetic differences in olive tree architecture and their effect on plant growth and fruiting was reported in Rosati et al. (2013), where the two cultivars most used in SHD orchards, i.e. Arbequina and Arbosana, were compared to 19 other cultivars, and in subsequent work comparing Arbequina to Frantoio (Rosati et al., 2018c). The results will be discussed in the following sections.

### 4.1 Trunk and branch architecture

Arbosana and Arbequina have lower trunk diameter growth compared to most other cultivars (Rosati et al., 2013), supporting the suggestion that such cultivars have lower vigor (Tous et al., 2006; Camposeo et al., 2008; Camposeo et al., 2022) and smaller trunks (Moutier, 2006). Reduced vigor in these cultivars is associated with early bearing (see section 2), but in this section we will analyze only architectural characteristics.

**Figure 1:**
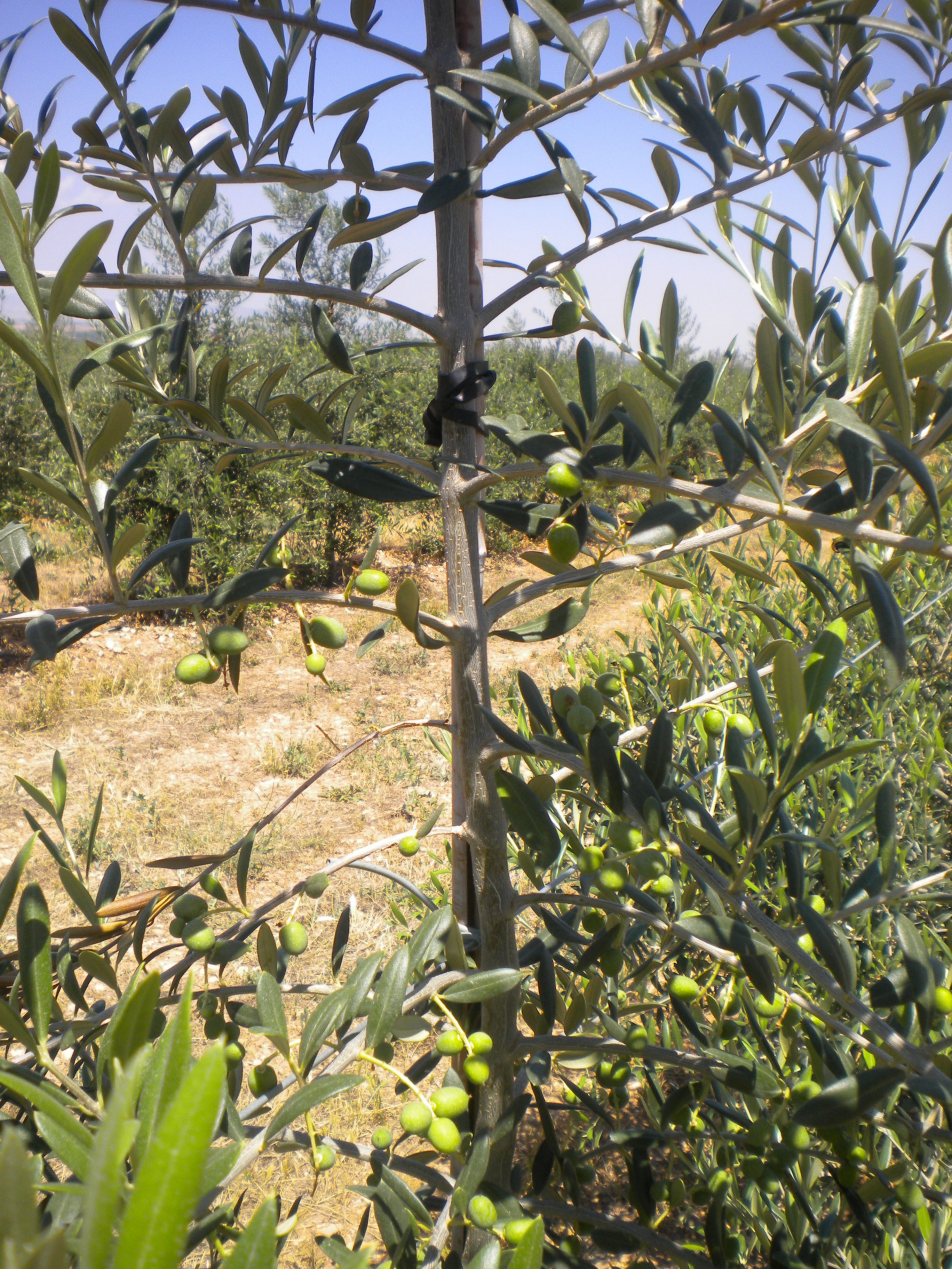
High branching frequency and density in Arbequina.

Arbequina and Arbosana also have higher branching frequency (i.e. number of branches per bud) and density (i.e. number of branches per unit of trunk of branch length) than most other cultivars (Rosati et al., 2013; **Figure 1**). The branching frequency of these cultivars was up to double that of some other cultivars. A higher branching frequency implies greater ability to fill a given canopy volume with fruiting sites (i.e. shoots) (Rosati et al., 2013; Rosati et al., 2018c), as reported also for other species (Lespinasse and Delort, 1986; Forshey et al., 1992; Lauri, 2007). Increasing branching frequency increases the fruiting sites per unit of biomass of supporting structures, making scale economy of the trunk and branches (Rosati et al., 2013; Rosati et al., 2018c). This scale economy is further increased by reduced trunk and branch diameter and growth. Combining these two aspects into a single parameter, denominated branching efficiency (i.e. number of branches per unit of trunk cross sectional area) segregated Arbequina and Arbosana from all other cultivars tested (Rosati et al., 2013), suggesting that these cultivars produce more fruiting sites per unit of trunk and branch mass, than any other cultivar. Arbequina and Arbosana do not differ from other cultivars for other architectural parameters of the canopy, like branch insertion angle (Rosati et al., 2013).

### 4.2 Shoot architecture

Shoot internode length does not differ between Arbequina and Arbosana and other cultivars Rosati et al. (2013). Therefore, while shorter internodes would increase canopy density and the number of potential fruiting sites per unit of canopy volume, this is not the feature that distinguishes those cultivars, from others non-suitable for SHD orchards. The same authors also found the following shoot characteristics. Internode length varies greatly with shoot length: longer shoots have longer internodes, but with no difference between Arbequina and Arbosana and all other cultivars. Instead, Arbequina and Arbosana differ from most cultivars in shoot diameter, having thinner shoots at equal shoot length. Shoot diameter is negatively correlated with branching frequency: cultivars that branch more, like Arbequina and Arbosana, also have thinner shoots, branches and trunks. Tus, this architectural pattern (i.e. thinner structures) is consistent from shoots to trunk. Shoot and branch insertion angle does not appear to differentiate Arbequina and Arbosana from other cultivars.

These findings indicate that olive cultivars differ in some architectural features, particularly in the branching pattern and in the diameter of wooden structures: cultivars range from low branching frequency and large diameters, to high branching frequency and thinner structures. In other fruit species, higher branching and small structure diameters result in more abundant bloom and fruiting, given that more shoots of lower vigor are produced for a given volume of canopy, increasing the number of productive sites (Bell, 1991). This is strategic in SHD orchards, where canopy volume is necessarily restricted to what fits into the harvester. Understanding the architectural features (and their genetics) allowing to maintain the canopy small and productive is essential for breeding and cultivar selection for SHD orchard, also allowing to reduce costs for pruning (Laurens et al., 2000).

## 5 Tree architecture and biomass partitioning

### 5.1 Shoot architecture and biomass partitioning

At the level of the shoot, the cost of the supporting woody structure (i.e. the shoot stem) is captured by the ratio of wood to leaf biomass: stem biomass/leaf biomass, also referred to as axialization (Lauri and Kelner, 2001). Analyzing this ratio has proved important in fruit production. Several studies discussed how shoot length (i.e. long vs. short, or spur, shoots) affects the ability of the tree to produce and support fruit (Johnson and Lakso, 1986a; Johnson and Lakso, 1986b). The wood to leaf biomass ratio is higher in long shoots (i.e. higher wood cost) than in short (spur) shoots (Lauri and Kelner, 2001). This reduces and delays carbon export to fruit, compared to spur shoots which invest proportionally less in stem (i.e. wood), and cease growing earlier, making them stronger and earlier sources (Hansen, 1977; Lakso, 1984; Sansavini and Corelli, 1992). In fact, yield often correlates with spur leaf area but not long-shoot leaf area (Sansavini and Corelli, 1992; Wünsche et al., 1996; Lakso et al., 1999). Hence, knowledge on the morphological variations among different shoot types and their physiological implications, particularly on fruiting ability, is both scientifically and practically important, as it can lead to improving management practices and yield (Normand et al., 2009).

While the wood to leaf biomass ratio affects the fruit production ability, fruit production, in turn, should affects the wood to leaf biomass ratio of the growing shoots. In fact, reproduction competes for resources with vegetative growth (see section 2), depressing it (Salazar-García et al., 1998; Stevenson and Shackel, 1998; Lauri and Térouanne, 1999; Costes et al., 2000; Berman and DeJong, 2003), including in olive (Monselise and Goldschmidt, 1982; Rallo and Suárez, 1989; Obeso, 2002; Connor and Fereres, 2005; Lavee, 2007; Dag et al., 2010; Castillo-Llanque and Rapoport, 2011). This generally results in shorter shoots in fruiting than to non-fruiting trees (Barlow, 1964; Avery, 1969; Avery, 1970) including in olive (Castillo-Llanque and Rapoport, 2011). If fruiting induces the formation of shorter shoots with earlier cessation of growth and lower wood to leaf biomass ratio, this should improve the canopy’s ability to support fruit production.

This was indeed demonstrated in a recent study on the effect of fruit load on shoot characteristics, across two olive cultivars with different fruit loads (Rosati et al., 2018c). These authors, working at the whole-tree level, found that fruit load decreased average shoot biomass, shoot leaf area, stem length and diameter, stem mass, internode length, and individual leaf mass, with single quantitative relationships across cultivars. Stem mass increased exponentially with stem length, due to the simultaneous increase in stem diameter. Internode length increased with shoot length. The simultaneous increase in shoot diameter and internode length, increased exponentially the amount of wood per leaf in longer shoots. A modest increase in individual leaf mass was not sufficient to compensate for this, and the wood to leaf biomass ratio increased by a factor of four, from short to long shoots. Considering that photosynthesis is mainly a function of leaf area rather than leaf mass, the wood to leaf biomass ratio would be better expressed in terms of leaf area rather than mass; however, specific leaf area did not vary across treatments, therefore differences in wood to leaf biomass ratios amounted to equivalent differences in wood biomass to leaf area ratio.

The data of Rosati et al. (2018c) showed that crop load strongly affects shoot length and, consequently, the shoot wood to leaf biomass ratio. At the highest fruit load, this ratio was as low as found in apple spurs, while in defruited trees, it was as high as in apple long shoots. Therefore, although the olive does not produce spurs or burses (Castillo-Llanque and Rapoport, 2011), shoots of different length have wood to leaf biomass ratios as different as found in apple short vs. long shoots. This suggests that olive short shoots are equivalent to apple spurs in terms of wood and leaf biomass costs and thus better able to sustain production. Since short shoots cease growth earlier than long shoot (Barlow, 1964; Avery, 1969; Avery, 1970), they are also likely to have earlier, in addition to greater potential for carbon export, as is the case in other species (Hansen, 1977; Lakso, 1984; Sansavini and Corelli, 1992; Lauri and Kelner, 2001). These findings support the suggestion that when analyzing the shoot architecture and the wood to leaf biomass ratio across different variables, such as different cultivars, shoot types, shoot ages, etc., it is necessary to account for fruit load and its effects on shoot length and architecture (Lauri and Kelner, 2001). Additionally, when studying shoot architecture, working with individual shoots may be inadequate to study the variability of traits across the whole population of shoots in the canopy.

In previous works, short vs. long shoots were compared (Barlow, 1964; Avery, 1969; Avery, 1970; Lauri and Kelner, 2001). Rosati et al. (2018c) showed instead that the wood to leaf biomass ratio varies with shoot length as a continuous function. Therefore, at least in olive, this ratio may not be a qualitative trait of different shoot types (i.e. spur vs. long), but rather a quantitative trait related to shoot length.

Although shorter shoots may be formed in response to high fruit loads (as a result of resource competition between shoots and fruit), their lower wood to leaf biomass ratio may be interpreted as a plant adaptation mechanism to support concurrent fruit growth. In fact, as an alternative to shorter shoots, the plant could react to increased resource competition from fruit with proportional reductions shoot number, while maintaining shoot length. However, this would reduce leaf mass (and area, and thus photosynthesis) proportionally to the reduction in the resources available for vegetative growth. Instead, producing more shoots, but shorter (lower wood to leaf biomass ratio), reduces shoot wood more than it reduces leaf area, with the advantages of earlier and greater carbon export towards fruit, as already discussed. This agrees with, and provides an explanation for, previous indications that competition with fruit reduces growth of woody structures more than leaf growth (Maggs, 1963; Cannell, 1985): this result is achieved, at least in part, by the formation of shorter shoots with lower wood to leaf biomass ratio.

Previous work indicated that short shoots are more likely to flower and fruit, whereas long shoots are more often vegetative (Bell, 1991). This may result from the different wood to leaf biomass ratio of the two shoot types, which affects the shoot carbon export ability in terms of timing and amount (Lauri and Térouanne, 1991; Lauri, 1992; Lauri and Kelner, 2001). In olive, however, this may not be the case. As an alternate bearing species, short shoots are typically formed during a year of high fruit load (i.e. an “on” year). The ability of these short shoots to flower and set fruit in the following year (i.e. “off” year) is low (Cimato and Fiorino, 1986; Fernández-Escobar et al., 1992; Ramos et al., 2000). On the other hand, during the “on” year, flowering and fruiting is abundant on the long shoots formed during the previous “off” year. In olive, therefore, shoot length and the consequent wood to leaf biomass ratio do not determine the subsequent fruiting level, which is related, instead, to the previous-year fruit load (Castillo-Llanque and Rapoport, 2011). Therefore, although short shoots have wood to leaf biomass ratios similar to burses and other short structures, these latter are specialized for fruiting, while the short shoots in olive are not specialized, and differ from longer shoots only for growing less in response to increased competition. Therefore, while their lower wood to leaf biomass ratio enhances concurrent fruit growth (occurring on previous-year shoots), it does not enhance their own flower induction and differentiation, which is decreased by the concurrent fruit growth.

### 5.2 Canopy architecture and biomass partitioning

The shoot wood to leaf biomass ratio concept can be applied at the whole-tree level, considering the whole-tree wood to leaf biomass ratio. In different species, there are cultivars differences in branching levels (Lespinasse and Delort, 1986; Forshey et al., 1992; Lauri, 2007) including in olive, where cultivars like Arbequina and Arbosana have the highest branching (Rosati et al., 2013; Rosati et al., 2018c). Higher branching results in a greater number of shoots per branch and greater number of branches per trunk and root. This implies that less wood is required to support the same number of shoots, resulting in lower wood to leaf biomass ratio at the whole-canopy level (Rosati et al., 2018c). A lower wood to leaf biomass ratio at the whole-tree level is likely to increase the canopy’s carbon export ability, as is the case at the shoot level. In fact, in young (i.e. small) trees, where self-shading is little, greater leaf area per unit of canopy biomass entails higher radiation interception and faster growth, as documented in grasses (Poorter and Pothmann, 1992). Lower whole-canopy wood to leaf biomass ratios allow to achieve autotrophy more rapidly in young plants of *Rubus alceifolius* (Baret et al., 2003).

## 6 Fruiting characteristics

Among the characteristics that distinguish the most used cultivars for SHD orchards is early bearing and a low alternate bearing tendency (Moutier et al., 2004; Moutier, 2006; Tous et al., 2006; Camposeo et al., 2008; Godini et al., 2011; Camposeo et al., 2022), as well as higher numbers of flowers and fruits per node (Rosati et al., 2013). However, fruit number is not the best parameter to assess the yield potential, as fruit size varies largely among cultivars (Barranco, 1999) and there is compensation between fruit number and size (Rosati et al., 2010). This compensation is determined by the different sink strength associated with different fruit size and cell number (Rosati et al., 2012; Rosati et al., 2020). Varietal differences in fruits size are already pre-determined at bloom in the form of ovary size differences (Rosati et al., 2009; Rosati et al., 2012), therefore the compensation between size and number must occur early, and affects both pistil abortion (Rosati et al., 2011) and fruit set (Rosati et al., 2010). Cultivars suitable for SHD orchards are also characterized by higher branching and lower wood to leaf biomass ratio, both at the shoot and the whole-tree level, as discussed in section 5. In fact, when comparing Arbequina and Arbosana to many other cultivars not suitable for SHD orchards, Rosati et al. (2013) found that some cultivars (e.g. Rosciola, Maurino) had flowering and fruiting parameters similar to them at the single shoot level, but not accompanied by high branching and smaller diameters of woody structures. On the other hand, other cultivars had similar branching qualities (e.g. Piantone di Mogliano, Piantone di Falerone), but insufficient fruiting. Only when both architectural and fruiting features were combined together, Arbequina and Arbosana were segregated from all other cultivars, suggesting that both features are required to achieve high yields in small canopy volumes, and thus suitability to SHD systems.

## 7 Conclusions

Olive tree growth appears to be mostly source limited and vegetative growth competes with fruit production. Therefore, yield and growth depend on partitioning of resource toward these two sinks. Defruiting makes trees grow more, while fruit presence slows down growth and produces shorter shoots. Short shoots have lower wood to leaf biomass ratio and thus greater and earlier ability to export carbon, which, in turn, supports fruit growth. However, in most cultivars, the short shoots formed during a year of abundant production will not flower and set fruit profusely the following year, setting off the alternate bearing cycle. Some cultivars, i.e. those most suitable for SHD systems, bear more fruit per node, thus allowing fruiting also on the short shoots formed in an “on” year. This reduces alternate bearing and increases fruiting efficiency by constantly producing shorter shoots with lower wood to leaf biomass ratio. However, these cultivars also have higher branching and thinner woody structures, both features increasing the leaf area and the number of fruiting sites per unit of wood biomass and per unit of canopy volume. Increased leaf area increases radiation capture and thus resource availability. Increasing the number of fruiting sites per unit of wood biomass saves resource investments in non-productive sinks (roots, trunk and branches), thus liberating resources which can be exported for fruit set and growth, increasing fruiting efficiency (resource partitioning towards fruit). Increased resource availability for fruit (from increased leaf area and reduced partitioning into woody structures) might in fact explain, at least in part, the greater flowering and fruit set ability of such cultivars.

**Figure 2:**
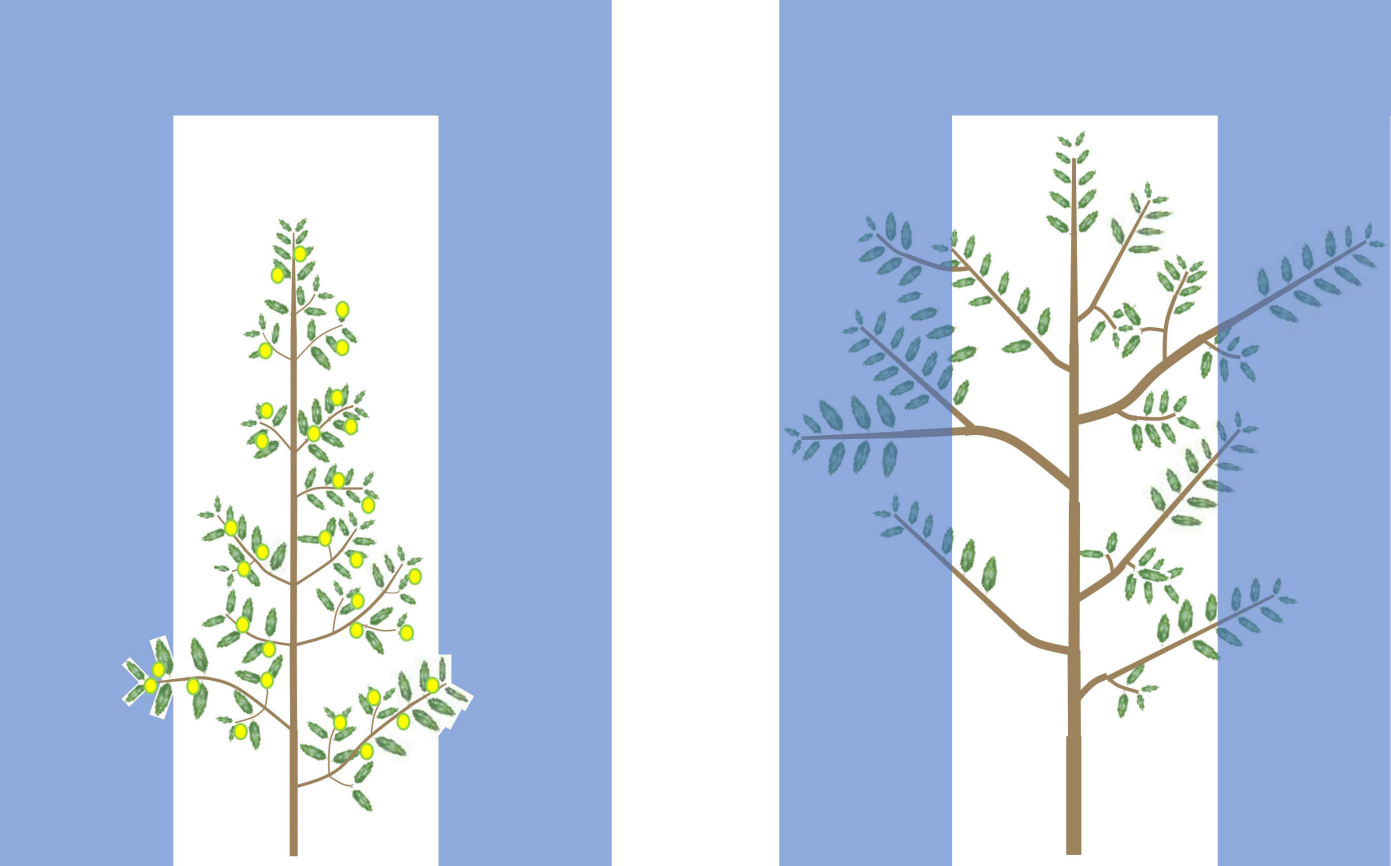
Olive cultivars suitable for high and super high density orchards have higher branching and thinner woody structures (left picture) than unsuitable cultivars (right picture). This increases the leaf area (and thus radiation capture and resource availability) and the number of fruiting sites per unit of woody biomass and per unit of canopy volume. This saves resource investments in non-productive sinks (roots, trunk and branches), thus liberating resources for fruit set and growth, possibly explaining, at least in part, the greater flowering and fruit set ability of such cultivars. Therefore, high branching reduces canopy volume directly, but also indirectly by increasing resources for fruiting, which, in turn, reduces vegetative growth, reducing canopy volume further. This reduces the pruning intensity needed to maintain the canopy within the allowed volume. Reduced pruning, in turn, allows greater fruiting, triggering a virtuous circle.

All of this suggests that possessing the right architectural characteristics is essential for cultivar breeding and selection for higher yield and yield efficiency, in intensive and, particularly, in super intensive systems where canopy volume is necessarily limited. Under these conditions, in fact, low branching not only reduces directly resource availability for fruit, but it also induces rapid growth outside the allowed volume, requiring intense pruning, which, in turn, reduces fruiting sites and thus fruiting. With lower fruiting, vigor increases further, requiring even more pruning, triggering a vicious circle of producing more vegetation and lower yields (i.e. low fruiting efficiency). High branching, instead, directly reduces canopy growth in terms of volume, but also liberates resources thus increasing fruiting, which, in turn, controls vigor further. With reduced vigor, less pruning is required, allowing greater fruiting, triggering a virtuous circle. These concepts are visualized and summarized in **Figure 2**.

## Author contributions

AR: Conceptualization, Funding acquisition, Investigation, Supervision, Visualization, Writing – original draft, Writing – review & editing. AP: Investigation, Writing – review & editing. EL: Investigation, Writing – review & editing. FF: Conceptualization, Investigation, Supervision, Writing – original draft, Writing – review & editing.

## References

[B1] Acebedo M. M. Cañete M. L. Cuevas J. (2000). Processes affecting fruit distribution and its quality in the canopy of olive trees. Adv. Hortic. Sci. 14, 169–175. doi: 10.1400/14060

[B2] Ainsworth E. A. Rogers A. Nelson R. Long S. P. (2004). Testing the “source–sink” hypothesis of down-regulation of photosynthesis in elevated [CO_2_] in the field with single gene substitutions in *Glycine max* . Agr. Forest. Meteorol. 122, 85–94. doi: 10.1016/j.agrformet.2003.09.002

[B3] Archbold D. D. Brown G. R. Cornelius P. L. (1987). Rootstock and in-row spacing effects on growth and yield of spur-type ‘Delicious’ and ‘Golden Delicious’ apple. J. Am. Soc Hortic. Sci. 112, 219–222. doi: 10.21273/JASHS.112.2.219

[B4] Atkinson C. J. Else M. A. (2003). “Enhancing harvest index in temperate fruit tree crops through the use of dwarfing rootstocks,” in Proceedings of International workshop on cocoa breeding for improved production systems, Accra, Ghana, (London, UK: INGENIC) 19-21 October 2003. 118–131.

[B5] Avery D. J. (1969). Comparisons of fruiting and deblossomed maiden apple trees, and of non-fruiting trees on a dwarfing and an invigorating rootstock. New Phytol. 68, 323–336. doi: 10.1111/j.1469-8137.1969.tb06444.x

[B6] Avery D. J. (1970). Effects of fruiting on the growth of apple trees on four rootstock varieties. New Phytol. 69, 19–30. doi: 10.1111/j.1469-8137.1970.tb04045.x

[B7] Bacelar E. A. Moutinho-Pereira J. M. Gonçalves B. C. Ferreira H. F. Correira C. M. (2007). Changes in growth, gas exchange, xylem hydraulic properties and water use efficiency of three olive cultivars under contrasting water availability regimes. Environ. Exp. Bot. 60, 183–192. doi: 10.1016/j.envexpbot.2006.10.003

[B8] Baldoni L. Fontanazza G. (1990). Preliminary results on olive clonal rootstocks behavior in the field. Acta Hortic. 286, 37–40. doi: 10.17660/ActaHortic.1990.286.2

[B9] Baret S. Nicolini E. Le Bourgeois T. Strasberg D. (2003). Developmental patterns of the invasive bramble (*Rubus alceifolius* Poiret, Rosaceae) in Réunion Island: an architectural and morphometric analysis. Ann. Bot. 91, 39–48. doi: 10.1093/aob/mcg006 12495918 PMC4240351

[B10] Barlow H. W. B. (1964). An interim report on a long-term experiment to assess the effect of cropping on apple tree growth. Annu. Rept. East Malling Res. Sta. 1963, 84–93.

[B11] Barranco D. (1997). “Variedades y patrones,” in El cultivo del olivo. Mundi Prensa. Eds. Barranco D. Fernández-Escobar R. Rallo L. (Madrid), 59–79.

[B12] Barranco D. (1999). “Variedades y patrones,” in El cultivo del olivo. Eds. Barranco D. Fernàndez-Escobar R. Rallo L. (Madrid, Spain: Ed. Mundi-Prensa), 63–89.

[B13] Barthélémy D. Caraglio Y. (2007). Plant architecture: a dynamic, multilevel and comprehensive approach to plant form, structure and ontogeny. Ann. Bot. 99, 375–407. doi: 10.1093/aob/mcl260 17218346 PMC2802949

[B14] Barthélémy D. Caraglio Y. Costes E. (1997). “Architecture, gradients morphogénétiques et âge physiologique chez les végétaux,” in Modélisation et simulation de l’architecture des végétaux. Eds. Bouchon J. Reffye P. Barthelemy D. (Paris: INRA éditions), 89–136.

[B15] Bell A. (1991). Plant form: An illustrated guide to flowering plant morphology (Oxford: University Press), 356 pp.

[B16] Berman M. E. DeJong T. M. (2003). Seasonal patterns of vegetative growth and competition with reproductive sinks in peach (*Prunus persica*). J. Hortic. Sci. Biotechnol. 78, 303–309. doi: 10.1080/14620316.2003.11511622

[B17] Bote A. D. Jan V. (2016). Branch growth dynamics, photosynthesis, yield and bean size distribution in response to fruit load manipulation in coffee trees. Trees 30, 1275–1285. doi: 10.1007/s00468-016-1365-x

[B18] Boussingault J. B. (1868). Agronomie, chimie agricole et physiologie. 2nd edn (Paris: Mallet Bachelier).

[B19] Butler J. (2014) New Olive Variety Launched by Agromillora. Olive Oil Times. Available at: https://www.oliveoiltimes.com/production/oliana-variety-launched-agromillora/39022 (Accessed 03 January 2024).

[B20] Camposeo S. Ferrara G. Palasciano M. Godini A. (2008). Varietal behaviour according to the superintensive oliveculture training system. Acta Hortic. 791, 271–274. doi: 10.17660/ActaHortic.2008.791.38

[B21] Camposeo S. Stellacci A. M. Romero Trigueros C. Alhajj Ali S. Vivaldi G. A. (2022). Different suitability of olive cultivars resistant to Xylella fastidiosa to the super-intensive planting system. Agronomy 12 (12), 3157. doi: 10.3390/agronomy12123157

[B22] Camposeo S. Vivaldi G. A. Montemurro C. Fanelli V. Cunill Canal M. (2021). Lecciana, a new low-vigour olive cultivar suitable for super high density orchards and for nutraceutical EVOO production. Agronomy 11 (11), 2154. doi: 10.3390/agronomy11112154

[B23] Cannell M. G. R. (1985). “Dry matter partitioning in tree crops,” in Attributes of trees as crop plants. Eds. Cannell M. G. R. Jackson J. E. (Midlothian, Great Britain: Inst. Terrestrial Ecology), 160–193.

[B24] Caraglio Y. Barthélémy D. (1997). “Revue critique des termes relatifs à la croissance et à la ramification des tiges des vègètaux vasculaires,” in Modélisation et simulation de l’Architecture des végétaux. Eds. Bouchon J. Reffye P. Barthélémy D. (Paris: INRA éditions), 11–88.

[B25] Caruso T. Campisi G. Marra F. P. Camposeo S. Vivaldi G. A. Proietti P.. (2014). Growth and yields of ‘Arbequina’ high-density planting systems in three different olive growing areas in Italy. Acta Hortic. 1057, 341–348. doi: 10.17660/ActaHortic.2014.1057.40

[B26] Castillo-Llanque F. Rapoport H. F. (2011). Relationship between reproductive behavior and new shoot development in 5-year-old branches of olive trees (*Olea europaea* L.). Trees 25, 823–832. doi: 10.1007/s00468-011-0558-6

[B27] Chandler W. H. Heinicke A. J. (1926). The effect of fruiting on the growth of Oldenburg apple trees. Proc. Am. Soc Hortic. Sci. 23, 36–46.

[B28] Cimato A. Fiorino P. (1986). Influence of fruit bearing on flower induction and differentiation in olive. Olea 17, 55–60.

[B29] Connor D. J. Fereres E. (2005). The physiology of adaptation and yield expression in olive. Hortic. Rev. 31, 155–229. doi: 10.1002/9780470650882.ch4

[B30] Cook N. C. Jacobs G. (1999). Suboptimal winter chilling impedes development of acrotony in apple shoots. HortScience 34, 1213–1216. doi: 10.21273/HORTSCI.34.7.1213

[B31] Costes E. Fournier D. Salles J. C. (2000). Changes in primary and secondary growth as influenced by crop load in ‘Fantasmet’ apricot trees. J. Hortic. Sci. Biotechnol. 75, 510–519. doi: 10.1080/14620316.2000.11511277

[B32] Costes E. Lauri P. E. Regnard J. L. (2006). Analyzing fruit tree architecture: implications for tree management and fruit production. Hortic. Rev. 32, 1–61. doi: 10.1002/9780470767986.ch1

[B33] Dag A. Bustan A. Avni A. Tzipori I. Lavee S. Riov J. (2010). Timing of fruit removal affects concurrent vegetative growth and subsequent return bloom and yield in olive (*Olea europaea* L.). Sci. Hortic. 123, 469–472. doi: 10.1016/j.scienta.2009.11.014

[B34] De Benedetto A. Jacoboni A. Venzi L. Pannelli G. (2003). Valutazione economica di un moderno impianto olivicolo nell’Italia centrale. Olivæ 95, 10–17.

[B35] DeJong T. M. (1986). Fruit effects on photosynthesis in *Prunus persica* . Physiol. Plant 66, 149–153. doi: 10.1111/j.1399-3054.1986.tb01248.x

[B36] DeJong T. M. (1999). Developmental and environmental control of dry-matter partitioning in peach. HortScience 34, 1037–1040. doi: 10.21273/HORTSCI.34.6.1037

[B37] Del Río C. García-Fernández M. D. Caballero J. M. (2002). Variability and classification of olive cultivars by their vigor. Acta Hortic. 586, 229–232. doi: 10.17660/ActaHortic.2002.586.43

[B38] Dichio B. Romano M. Nuzzo V. Xiloyannis C. (2002). Soil water availability and relationship between canopy and roots in young olive trees (cv Coratina). Acta Hortic. 586, 255–258. doi: 10.17660/ActaHortic.2002.586.48

[B39] Díez C. M. Moral J. Cabello D. Morello P. Rallo L. Barranco D. (2016). Cultivar and tree density as key factors in the long-term performance of super high-density olive orchards. Front. Plant Sci. 7. doi: 10.3389/fpls.2016.01226 PMC4993835 27602035

[B40] Di Vaio C. Marallo N. Marino G. Caruso T. (2013). Effect of water stress on dry matter accumulation and partitioning in pot-grown olive trees (cv Leccino and Racioppella). Sci. Hortic. 164, 155–159. doi: 10.1016/j.scienta.2013.09.008

[B41] Di Vaio C. Marra F. P. Scaglione G. La Mantia M. Caruso T. (2012). The effect of different vigour olive clones on growth, dry matter partitioning and gas exchange under water deficit. Sci. Hortic. 134, 72–78. doi: 10.1016/j.scienta.2011.11.001

[B42] Donadio L. C. Lederman I. E. Roberto S. R. Stucchi E. S. (2019). Dwarfing-canopy and rootstock cultivars for fruit trees. Rev. Bras. Frutic. 41, e997. doi: 10.1590/0100-29452019997

[B43] Donald C. M. (1962). In search of yield. J. Aust. Inst. Agric. Sci. 28, 171–178.

[B44] Egli D. B. Bruening W. P. (2003). Increasing sink size does not increase photosynthesis during seed filling in soybean. Eur. J. Agron. 19, 289–298. doi: 10.1016/S1161-0301(02)00074-6

[B45] Elfving D. C. (1988). Economic effects of excessive vegetative growth in deciduous fruit trees. HortScience 23, 461–463. doi: 10.21273/HORTSCI.23.3.461

[B46] Embree C. G. Myra M. T. D. Nichols D. S. Wright A. H. (2007). Effect of blossom density and crop load on growth, fruit quality, and return bloom in ‘Honeycrisp’ apple. HortScience 42, 1622–1625. doi: 10.21273/HORTSCI.42.7.1622

[B47] Evans L. T. (1976). Physiological adaptation to performance as crop plants. Phil. Trans. R. Soc Lond. B 275, 71–83. doi: 10.1098/rstb.1976.0071

[B48] Famiani F. Cinosi N. Paoletti A. Farinelli D. Rosati A. Lodolini E. M. (2022). Deflowering as a tool to accelerate growth of young trees in both intensive and super-high-density olive orchards. Agronomy 12, 2319. doi: 10.3390/agronomy12102319

[B49] Famiani F. Farinelli D. Gardi T. Rosati A. (2019). The cost of flowering in olive (*Olea europaea* L.). Sci. Hortic. 252, 268–273. doi: 10.1016/j.scienta.2019.03.008

[B50] Farinelli D. Tombesi S. (2015). Performance and oil quality of ‘Arbequina’ and four Italian olive cultivars under super high density hedgerow planting system cultivated in central Italy. Sci. Hortic. 192, 97–107. doi: 10.1016/j.scienta.2015.04.035

[B51] Fatichi S. Leuzinger S. Körner C. (2014). Moving beyond photosynthesis: from carbon source to sink-driven vegetation modeling. New Phytol. 201, 1086–1095. doi: 10.1111/nph.12614 24261587

[B52] Fernández-Escobar R. Benlloch M. Navarro C. Martin G. C. (1992). The time of floral induction in the olive. J. Am. Soc Hortic. Sci. 117, 304–307. doi: 10.21273/JASHS.117.2.304

[B53] Fischer G. Almanza-Merchán P. J. Ramírez F. (2012). Source-sink relationships in fruit species: A review. Rev. Colomb. Cienc. Hortíc. 6, 238–253. doi: 10.17584/rcch.2012v6i2.1980

[B54] Forshey C. G. Elfving D. C. (1989). The relationship between vegetative growth and fruiting in apple trees. Hortic. Rev. 11, 229–287. doi: 10.1002/9781118060841.ch7

[B55] Forshey C. G. Elfving D. C. Stebbins R. L. (1992). Training and pruning apple and pear trees (Alexandria, Virginia: American Society for Horticultural Science).

[B56] Forshey C. G. McKee M. W. (1970). Production efficiency of a large and a small ‘McIntosh’ apple tree. HortScience 5, 164–165. doi: 10.21273/HORTSCI.5.3.164

[B57] Franck N. Vaast P. Génard M. Dauzat J. (2006). Soluble sugars mediate sink feedback down-regulation of leaf photosynthesis in field-grown *Coffea arabica* . Tree Physiol. 26, 517–525. doi: 10.1093/treephys/26.4.517 16414930

[B58] Geisler D. Ferree D. C. (1984). The influence of root pruning on water relations, net photosynthesis, and growth of young ‘Golden Delicious’ apple trees. J. Am. Soc Hortic. Sci. 109, 827–831. doi: 10.21273/JASHS.109.6.827

[B59] Godini A. Vivaldi G. A. Camposeo S. (2011). Olive cultivars field-tested in super-high-density system in southern Italy. Calif. Agr. 65, 39–40. doi: 10.3733/ca.v065n01p39

[B60] Grossman Y. L. DeJong T. M. (1995a). Maximum fruit growth potential and seasonal patterns of resource dynamics during peach growth. Ann. Bot. 75, 553–560. doi: 10.1006/anbo.1995.1058

[B61] Grossman Y. L. DeJong T. M. (1995b). Maximum vegetative growth potential and seasonal patterns of resource dynamics during peach growth. Ann. Bot. 76, 473–482. doi: 10.1006/anbo.1995.1122

[B62] Gucci R. Corelli Grappadelli L. Tustin S. Ravaglia G. (1995). The effect of defruiting at different stages of fruit development on leaf photosynthesis of “Golden Delicious” apple. Tree Physiol. 15, 35–40. doi: 10.1093/treephys/15.1.35 14966009

[B63] Gucci R. Xiloyannis C. Flore J. A. (1991). Gas exchange parameters, water relations and carbohydrate partitioning in leaves of field-grown *Prunus domestica* following fruit removal. Physiol. Plant 83, 497–505. doi: 10.1111/j.1399-3054.1991.tb00126.x

[B64] Guerrero-Casado J. Carpio A. J. Tortosa F. S. Villanueva A. J. (2021). Environmental challenges of intensive woody crops: The case of super high-density olive groves. Sci. Total Environ. 798, 149212. doi: 10.1016/j.scitotenv.2021.149212 34315052

[B65] Hallé F. Oldeman R. A. A. (1970). Essai sur l’architecture et la dynamique de croissance des arbres tropicaux (Paris: Masson).

[B66] Hallé F. Oldeman R. A. A. Tomlinson P. B. (1978). Tropical trees and forests: an architectural analysis (Berlin-Heidelberg-New York: Springer-Verlag).

[B67] Hammami S. B. M. León L. Rapoport H. F. de la Rosa R. (2011). Early growth habit and vigour parameters in olive seedlings. Sci. Hortic. 129, 761–768. doi: 10.1016/j.scienta.2011.05.038

[B68] Hammami S. B. M. León L. Rapoport H. F. de la Rosa R. (2021). A new approach for early selection of short juvenile period in olive progenies. Sci. Hortic. 281, 109993. doi: 10.1016/j.scienta.2021.109993

[B69] Hansen P. (1977). “Carbohydrate allocation,” in Environmental effects on crop physiology. Eds. Landsberg J. J. Cutting C. V. (London: Academic Press), 247–258.

[B70] Haouari A. Van Labeke M. C. Chehab H. Ben Meriem F. Steppe K. Braham M. (2011). Effect of leaf-to-fruit ratio and girdling on gas exchanges, fruit growth and carbohydrate contents at different stages of fruit development of *Olea europaea* L. ‘Picholine’. Acta Hortic. 924, 77–81. doi: 10.17660/ActaHortic.2011.924.8

[B71] Hasegawa S. Takeda H. (2001). Functional specialization of current shoots as a reproductive strategy in Japanese alder (*Alnus hirsuta* var. *sibirica*). Can. J. Bot. 79, 38–48. doi: 10.1139/b00-143

[B72] Iglesias D. J. Lliso I. Tadeo F. R. Talon M. (2002). Regulation of photosynthesis through source: sink imbalance in citrus is mediated by carbohydrate content in leaves. Physiol. Plant 116, 563–572. doi: 10.1034/j.1399-3054.2002.1160416.x

[B73] Jerie P. H. Van den Ende B. Dann I. R. (1989). Managing tree vigour and fruitfulness in deciduous orchards. Acta Hortic. 240, 127–134. doi: 10.17660/ActaHortic.1989.240.21

[B74] Johnson R. S. Lakso A. N. (1986a). Carbon balance model of a growing apple shoot: I. Development of the model. J. Am. Soc Hortic. Sci. 111, 160–164. doi: 10.21273/JASHS.111.2.160

[B75] Johnson R. S. Lakso A. N. (1986b). Carbon balance model of a growing apple shoot: II. Simulated effects of light and temperature on long and short shoots. J. Am. Soc Hortic. Sci. 111, 164–169. doi: 10.21273/JASHS.111.2.164

[B76] Koumanov K. S. Tsareva I. N. (2017). Intensive sweet cherry production: potential, “bottlenecks”, perspectives. Acta Hortic. 1161, 103–110. doi: 10.17660/ActaHortic.2017.1161.18

[B77] Kramer P. J. Kozlowski T. T. (1979). The physiology of woody plants (New York: Academic Press).

[B78] Labuschagné I. F. Louw J. H. Schmidt K. Sadie A. (2003). Selection for increased budbreak in apple. J. Am. Soc Hortic. Sci. 128, 363–373. doi: 10.21273/JASHS.128.3.0363

[B79] Lakso A. N. (1984). Leaf area development patterns in young pruned and unpruned apple trees. J. Am. Soc Hortic. Sci. 109, 861–865. doi: 10.21273/JASHS.109.6.861

[B80] Lakso A. N. Flore J. A. (2003). “Carbohydrate partitioning and plant growth,” in Concise encyclopedia of temperate tree fruit. Eds. Baugher T. A. Singh S. (New York: Food Products Press), 21–30.

[B81] Lakso A. N. Wünsche J. N. Palmer J. W. Corelli Grappadelli L. (1999). Measurement and modeling of carbon balance of the apple tree. HortScience 34, 1040–1047. doi: 10.21273/HORTSCI.34.6.1040

[B82] Laurens F. Audergon J. M. Claverie J. Duval H. Germain É. Kervella J.. (2000). Integration of architectural types in French programs of ligneous fruit species genetic improvement. Fruits 55, 141–152.

[B83] Lauri P.É. (1992). Données sur le contexte végétatif lié à la floraison chez le cerisier (*Prunus avium*). Can. J. Bot. 70, 1848–1859. doi: 10.1139/b92-229

[B84] Lauri P.É. (2007). Differentiation and growth traits associated with acrotony in the apple tree (*Malus* × *domestica*, Rosaceae). Am. J. Bot. 94, 1273–1281. doi: 10.3732/ajb.94.8.1273 21636493

[B85] Lauri P.É. Kelner J. J. (2001). Shoot type demography and dry matter partitioning: a morphometric approach in apple (*Malus × domestica*). Can. J. Bot. 79, 1270–1273. doi: 10.1139/b01-113

[B86] Lauri P.É. Lespinasse J. M. (2001). Genotype of apple trees affects growth and fruiting responses to shoot bending at various times of year. J. Am. Soc Hortic. Sci. 126, 169–174. doi: 10.21273/JASHS.126.2.169

[B87] Lauri P.É. Térouanne É. (1991). Éléments pour une approche morphométrique de la croissance végétale et de la floraison: le cas d’espèces tropicales du modèle de Leeuwenberg. Can. J. Bot. 69, 2095–2112. doi: 10.1139/b91-264

[B88] Lauri P.É. Térouanne É. (1999). Effects of inflorescence removal on the fruit set of the remaining inflorescences and development of the laterals on one year old apple (*Malus domestica* Borkh.) branches. J. Hortic. Sci. Biotechnol. 74, 110–117. doi: 10.1080/14620316.1999.11511082

[B89] Lavee S. (2007). Biennial bearing in olive (*Olea europaea*). Ann. Ser. His. Nat. 17, 101–112.

[B90] Layne D. R. Flore J. A. (1995). End-product inhibition of photosynthesis in *Prunus cerasus* L. @ in response to whole-plant source-sink manipulation. J. Am. Soc Hortic. Sci. 120, 583–599. doi: 10.21273/JASHS.120.4.583

[B91] León Moreno L. (2007). ‘Chiquitita’: una variedad para olivares en seto. Phytoma Espana 190, 32–33.

[B92] Lespinasse J. M. Delort J. F. (1986). Apple tree management in vertical axis: appraisal after ten years of experiments. Acta Hortic. 160, 120–155. doi: 10.17660/ActaHortic.1986.160.15

[B93] Li W. D. Duan W. Fan P. G. Yan S. T. Li S. H. (2007). Photosynthesis in response to sink—source activity and in relation to end products and activities of metabolic enzymes in peach trees. Tree Physiol. 27, 1307–1318. doi: 10.1093/treephys/27.9.1307 17545130

[B94] Loomis R. S. (1983). “Productivity of agricultural systems,” in Encyclopedia of Plant Physiology, vol. 12D . Eds. Lange O. L. Nobel P. S. Osmond C. B. Ziegler H. (Berlin, Heidelberg, New York: Springer-Verlag), 151–172.

[B95] Maggs D. H. (1963). The reduction in growth of apple trees brought about by fruiting. J. Hortic. Sci. 38, 119–128. doi: 10.1080/00221589.1963.11514065

[B96] Matsuda R. Suzuki K. Nakano A. Higashide T. Takaichi M. (2011). Responses of leaf photosynthesis and plant growth to altered source–sink balance in a Japanese and a Dutch tomato cultivar. Sci. Hortic. 127, 520–527. doi: 10.1016/j.scienta.2010.12.008

[B97] McCormick A. J. Cramer M. D. Watt D. A. (2006). Sink strength regulates photosynthesis in sugarcane. New Phytol. 171, 759–770. doi: 10.1111/j.1469-8137.2006.01785.x 16918547

[B98] Mitchell P. D. Chalmers D. J. (1983). A comparison of microjet and point emitter (trickle) irrigation in the establishment of a high-density peach orchard. HortScience 18, 472–474. doi: 10.21273/HORTSCI.18.4.472

[B99] Mitchell P. D. Van de Ende B. Jerie P. H. Chalmers D. J. (1989). Responses of ‘Bartlett’ pear to withholding irrigation, regulated deficit irrigation, and tree spacing. J. Am. Soc Hortic. Sci. 114, 15–19. doi: 10.21273/JASHS.114.1.15

[B100] Monselise S. Goldschmidt E. E. (1982). Alternate bearing in fruit trees. Hortic. Rev. 4, 128–173. doi: 10.1002/9781118060773.ch5

[B101] Moutier N. (2006). Olive tree architecture: different levels of approach. Olea 25, 33–35.

[B102] Moutier N. Garcia G. Lauri P.É. (2004). Shoot architecture of the olive tree: effect of cultivar on the number and distribution of vegetative and reproductive organs on branches. Acta Hortic. 636, 689–694. doi: 10.17660/ActaHortic.2004.636.86

[B103] Moutier N. Garcia G. Lauri P.É. (2008). Natural vegetative development and fruit production of olive trees. Acta Hortic. 791, 351–355. doi: 10.17660/ActaHortic.2008.791.50

[B104] Mulas M. Caddeo C. Bandino G. Sedda P. (2011). Shoot pruning and treatment with hexaconazole or urea to increase fruit-set in olive. Acta Hortic. 924, 233–240. doi: 10.17660/ActaHortic.2011.924.29

[B105] Muller B. Pantin F. Génard M. Turc O. Freixes S. Piques M.. (2011). Water deficits uncouple growth from photosynthesis, increase C content, and modify the relationships between C and growth in sink organs. J. Exp. Bot. 62, 1715–1729. doi: 10.1093/jxb/erq438 21239376

[B106] Naor A. Flaishman M. Stern R. Moshe A. Erez A. (2003). Temperature effects on dormancy completion of vegetative buds in apple. J. Am. Soc Hortic. Sci. 128, 636–641. doi: 10.21273/JASHS.128.5.0636

[B107] Nebauer S. G. Renau-Morata B. Guardiola J. L. Molina R. V. (2011). Photosynthesis down-regulation precedes carbohydrate accumulation under sink limitation in *Citrus* . Tree Physiol. 31, 169–177. doi: 10.1093/treephys/tpq103 21367744

[B108] Normand F. Bello A. K. P. Trottier C. Lauri P.É. (2009). Is axis position within tree architecture a determinant of axis morphology, branching, flowering and fruiting? An essay in mango. Ann. Bot. 103, 1325–1336. doi: 10.1093/aob/mcp079 19349282 PMC2685314

[B109] Obeso J. R. (2002). The cost of reproduction in plants. New Phytol. 155, 321–348. doi: 10.1046/j.1469-8137.2002.00477.x 33873312

[B110] Oldeman R. A. A. (1974). L’architecture de la forêt Guyanaise. Mémoire 73, O.R.S.T.O.M., Paris. 204 pp.

[B111] Palacio S. Hoch G. Sala A. Körner C. Millard P. (2014). Does carbon storage limit tree growth? New Phytol. 201, 1096–1100. doi: 10.1111/nph.12602 24172023

[B112] Pan Y. Lu Z. Lu J. Li X. Cong R. Ren T. (2017). Effects of low sink demand on leaf photosynthesis under potassium deficiency. Plant Physiol. Biochem. 113, 110–121. doi: 10.1016/j.plaphy.2017.01.027 28196349

[B113] Pannelli G. Famiani F. Rugini E. (1992). Effects of a change in ploidy level on anatomical, cytological, reproductive and growth performance changes and polyamine contents, in mutants of gamma irradiated olive plants. Acta Hortic. 317, 209–218. doi: 10.17660/ActaHortic.1992.317.25

[B114] Pannelli G. Rosati S. Rugini E. (2002). The effect of clonal rootstocks on frost tolerance and on some aspects of plant behaviour in Moraiolo and S. Felice olive cultivars. Acta Hortic. 586, 247–250. doi: 10.17660/ActaHortic.2002.586.46

[B115] Paoletti A. Cinosi N. Lodolini E. M. Famiani F. Rosati A. (2023). Effects of root constriction on vegetative growth and yield efficiency in young trees of a low- and a high-vigor olive cultivar. Trees 37, 1179–1187. doi: 10.1007/s00468-023-02416-2

[B116] Paoletti A. Rosati A. Famiani F. (2021). Effects of cultivar, fruit presence and tree age on whole-plant dry matter partitioning in young olive trees. Heliyon 7, e06949. doi: 10.1016/j.heliyon.2021.e06949 34013085 PMC8113714

[B117] Patrick J. W. (1988). Assimilate partitioning in relation to crop productivity. HortScience 23, 33–40. doi: 10.21273/HORTSCI.23.1.33b

[B118] Paul M. J. Foyer C. H. (2001). Sink regulation of photosynthesis. J. Exp. Bot. 52, 1383–1400. doi: 10.1093/jexbot/52.360.1383 11457898

[B119] Pieters A. J. Paul M. J. Lawlor D. W. (2001). Low sink demand limits photosynthesis under P_i_ deficiency. J. Exp. Bot. 52, 1083–1091. doi: 10.1093/jexbot/52.358.1083 11432924

[B120] Poorter H. Pothmann P. (1992). Growth and carbon economy of a fast-growing and a slow-growing grass species as dependent on ontogeny. New Phytol. 120, 159–166. doi: 10.1111/j.1469-8137.1992.tb01069.x

[B121] Preston A. P. (1958). Apple rootstock studies: thirty-five years’ results with Lane’s Prince Albert on clonal rootstocks. J. Hortic. Sci. 33, 29–38. doi: 10.1080/00221589.1958.11513911

[B122] Proietti P. (2000). Effect of fruiting on leaf gas exchange in olive (*Olea europaea* L.). Photosynthetica 38, 397–402. doi: 10.1023/A:1010973520871

[B123] Proietti P. Nasini L. Famiani F. (2006). Effect of different leaf-to-fruit ratio on photosynthesis and fruit growth in olive (*Olea europaea* L.). Photosynthetica 44, 275–285. doi: 10.1007/s11099-006-0019-4

[B124] Proietti P. Tombesi A. (1990). Effect of girdling on photosynthetic activity in olive leaves. Acta Hortic. 286, 215–218. doi: 10.17660/ActaHortic.1990.286.44

[B125] Proietti P. Tombesi A. (1996). Translocation of assimilates and source-sink influences on productive characteristics of the olive tree. Adv. Hortic. Sci. 10, 11–14. doi: 10.1400/75282

[B126] Quentin A. G. Close D. C. Hennen L. M. H. P. Pinkard E. A. (2014). Down-regulation of photosynthesis via sink limitation is linked to foliar soluble sugar content in high-and low-yielding varieties of sweet cherry. Acta Hortic. 1058, 251–256. doi: 10.17660/ActaHortic.2014.1058.42

[B127] Rallo L. Barranco D. de la Rosa R. León L. (2007). El programa de mejora genetica de Cordoba. Phytoma Espana 190, 22–31.

[B128] Rallo L. Barranco D. de la Rosa R. León L. (2008). ‘Chiquitita’olive. HortScience 43 (2), 529–531. doi: 10.21273/HORTSCI.43.2.529

[B129] Rallo L. Suárez M. P. (1989). Seasonal distribution of dry matter within the olive fruit-bearing limb. Adv. Hortic. Sci. 3, 55–59. doi: 10.1400/13932

[B130] Ramos A. Rallo L. Rapoport H. F. (2000). Effect of the bearing condition of the tree and defoliation on the dormancy onset and release of olive buds. Acta Hortic. 515, 297–304. doi: 10.17660/ActaHortic.2000.515.37

[B131] Ribeiro R. V. MaChado E. C. Magalhães Filho J. R. Lobo A. K. M. Martins M. O. Silveira J. A. G.. (2017). Increased sink strength offsets the inhibitory effect of sucrose on sugarcane photosynthesis. J. Plant Physiol. 208, 61–69. doi: 10.1016/j.jplph.2016.11.005 27889522

[B132] Rosati A. Caporali S. Hammami S. B. M. Moreno-Alìas I. Paoletti A. Rapoport H. F. (2012). Tissue size and cell number in the olive (*Olea europaea*) ovary determine tissue growth and partitioning in the fruit. Func. Plant Biol. 39, 580–587. doi: 10.1071/FP12114 32480810

[B133] Rosati A. Caporali S. Hammami S. B. Moreno-Alías I. Rapoport H. (2020). Fruit growth and sink strength in olive (Olea europaea) are related to cell number, not to tissue size. Funct. Plant Biol. 47 (12), 1098–1104. doi: 10.1071/FP20076 32669193

[B134] Rosati A. Caporali S. Paoletti A. Famiani F. (2011). Pistil abortion is related to ovary mass in olive (*Olea europaea* L.). Sci. Hortic. 127, 515–519. doi: 10.1016/j.scienta.2010.12.002

[B135] Rosati A. Paoletti A. Al Hariri R. Famiani F. (2018c). Fruit production and branching density affect shoot and whole-tree wood to leaf biomass ratio in olive. Tree Physiol. 38, 1278–1285. doi: 10.1093/treephys/tpy009 29452417

[B136] Rosati A. Paoletti A. Al Hariri R. Morelli A. Famiani F. (2018a). Partitioning of dry matter into fruit explains cultivar differences in vigor in young olive (*Olea europaea* L.) trees. HortScience 53, 491–495. doi: 10.21273/HORTSCI12739-17

[B137] Rosati A. Paoletti A. Al Hariri R. Morelli A. Famiani F. (2018b). Resource investments in reproductive growth proportionately limit investments in whole-tree vegetative growth in young olive trees with varying crop loads. Tree Physiol. 38, 1267–1277. doi: 10.1093/treephys/tpy011 29474732

[B138] Rosati A. Paoletti A. Caporali S. Perri E. (2013). The role of tree architecture in super high density olive orchards. Sci. Hortic. 161, 24–29. doi: 10.1016/j.scienta.2013.06.044

[B139] Rosati A. Paoletti A. Pannelli G. Famiani F. (2017). Growth is inversely correlated with yield efficiency across cultivars in young olive (*Olea europaea* L.) trees. HortScience 52, 1525–1529. doi: 10.21273/HORTSCI12321-17

[B140] Rosati A. Zipanćič M. Caporali S. Padula G. (2009). Fruit weight is related to ovary weight in olive (*Olea europaea* L.). Sci. Hortic. 122, 399–403. doi: 10.1016/j.scienta.2009.05.034

[B141] Rosati A. Zipanćič M. Caporali S. Paoletti A. (2010). Fruit set is inversely related to flower and fruit weight in olive (*Olea europaea* L.). Sci. Hortic. 126, 200–204. doi: 10.1016/j.scienta.2010.07.010

[B142] Rugini E. Pannelli G. (1993). Preliminary results on increasing fruit set in olives (*Olea europaea* L.) by chemical and mechanical treatments. Acta Hortic. 329, 209–210. doi: 10.17660/ActaHortic.1993.329.45

[B143] Saa S. Brown P. H. (2014). Fruit presence negatively affects photosynthesis by reducing leaf nitrogen in almond. Funct. Plant Biol. 41, 884–891. doi: 10.1071/FP13343 32481042

[B144] Salazar-García S. Lord E. M. Lovatt C. J. (1998). Inflorescence and flower development of the ‘Hass’ avocado (*Persea americana* Mill.) during ‘‘on’’ and ‘‘off’’ crop years. J. Am. Soc Hortic. Sci. 123, 537–544. doi: 10.21273/JASHS.123.4.537

[B145] Sansavini S. Corelli L. (1992). Canopy efficiency of apple as affected by microclimatic factors and tree structure. Acta Hortic. 322, 69–78. doi: 10.17660/ActaHortic.1992.322.7

[B146] Sawada S. Hayakawa T. Fukushi K. Kasai M. (1986). Influence of carbohydrates on photosynthesis in single, rooted soybean leaves used as a source-sink model. Plant Cell Physiol. 27, 591–600. doi: 10.1093/oxfordjournals.pcp.a077138

[B147] Scariano L. Lo Bianco R. Di Marco L. Policarpo M. (2008). Dynamics of dry matter partitioning in young ‘Nocellara del Belice’ olive trees. Acta Hortic. 791, 397–401. doi: 10.17660/ActaHortic.2008.791.58

[B148] Sonnoli A. (2001). Une nouvelle variété d’olivier à dimensions réduites. Olivae 88, 46–49.

[B149] Spurr S. H. Barnes B. V. (1980). Forest ecology. 3rd edn (New York: Wiley).

[B150] Stevenson M. T. Shackel K. A. (1998). Alternate bearing in pistachio as a masting phenomenon: construction cost of reproduction versus vegetative growth and storage. J. Am. Soc Hortic. Sci. 123, 1069–1075. doi: 10.21273/JASHS.123.6.1069

[B151] Stitt M. (1991). Rising CO_2_ levels and their potential significance for carbon flow in photosynthetic cells. Plant Cell Environ. 14, 741–762. doi: 10.1111/j.1365-3040.1991.tb01440.x

[B152] Tognetti R. Costagli G. Minnocci A. Gucci R. (2002). Stomatal behavior and water use efficiency in two cultivars of *Olea europaea* L. Agr. Med. 132, 90–97.

[B153] Tombesi S. Farinelli D. (2016). Trunk constriction effects vegetative vigor and yield efficiency in olive tree (*Olea europaea* L.). J. Agric. Sci. Technol. 18, 1667–1680.

[B154] Tous J. Romero A. Hermoso J. F. (2006). “High density planting systems, mechanisation and crop management in olive,” in Proceedings of the II International Seminar “Olivebiotech 2006”, Mazara del Vallo (TP), Italy, 5-10 November. 423–430.

[B155] Tous J. Romero A. Hermoso J. F. Msallem M. Larbi A. (2014). Olive orchard design and mechanization: Present and future. Acta Hortic. 1057, 231–246. doi: 10.17660/ActaHortic.2014.1057.27

[B156] Tous J. Romero A. Plana J. Baiges F. (1999). Planting density trial with ‘Arbequina’ olive cultivar in Catalonia (Spain). Acta Hortic. 474, 177–180. doi: 10.17660/ActaHortic.1999.474.34

[B157] Troncoso A. Liñán J. Prieto J. Cantos M. (1990). Influence of different olive rootstocks on growth and production of ‘Gordal Sevillana’. Acta Hortic. 286, 133–136. doi: 10.17660/ActaHortic.1990.286.26

[B158] Verheij E. W. M. (1972). Competition in apple as influenced by Alar sprays, fruiting, pruning and tree spacing (Wageningen: Meded. Landbouwhog), 72-4, l–54.

[B159] Villalobos F. J. Testi L. Hidalgo J. Pastor M. Orgaz F. (2006). Modelling potential growth and yield of olive (*Olea europaea* L.) canopies. Eur. J. Agron. 24, 296–303. doi: 10.1016/j.eja.2005.10.008

[B160] Vivaldi G. A. Strippoli G. Pascuzzi S. Stellacci A. M. Camposeo S. (2015). Olive genotypes cultivated in an adult high-density orchard respond differently to canopy restraining by mechanical and manual pruning. Scientia Hortic. 192, 391–399. doi: 10.1016/j.scienta.2015.06.004

[B161] Williams M. W. Curry E. A. Greene G. M. (1986). Chemical control of vegetative growth of pome and stone fruit trees with GA biosynthesis inhibitors. Acta Hortic. 179, 453–458. doi: 10.17660/ActaHortic.1986.179.70

[B162] Wünsche J. N. Ferguson I. B. (2005). Crop load interactions in apple. Hortic. Rev. 31, 231–290. doi: 10.1002/9780470650882.ch5

[B163] Wünsche J. N. Lakso A. N. Robinson T. L. Lenz F. Denning S. S. (1996). The bases of productivity in apple production systems: the role of light interception by different shoot types. J. Am. Soc Hortic. Sci. 121, 886–893. doi: 10.21273/JASHS.121.5.886

[B164] Xiloyannis C. Dichio B. Nuzzo V. Celano G. (1999). Defense strategies of olive against water stress. Acta Hortic. 474, 423–426. doi: 10.17660/ActaHortic.1999.474.86

[B165] Zhang W. W. Fu X. Z. Peng L. Z. Ling L. L. Cao L. Ma X. H.. (2013). Effects of sink demand and nutrient status on leaf photosynthesis of spring-cycle shoot in ‘Newhall’ navel orange under natural field conditions. Sci. Hortic. 150, 80–85. doi: 10.1016/j.scienta.2012.10.031

